# Resistance to cancer immunotherapy in metastatic renal cell carcinoma

**DOI:** 10.20517/cdr.2020.16

**Published:** 2020-07-02

**Authors:** Marco Moreira, Cedric Pobel, Nicolas Epaillard, Audrey Simonaggio, Stéphane Oudard, Yann-Alexandre Vano

**Affiliations:** ^1^Centre de Recherche des Cordeliers, INSERM, Université de Paris, Sorbonne Université, Paris F-75006, France.; ^2^Department of Medical Oncology, European Hospital Georges Pompidou - APHP, Paris 75015, France.; ^3^University François Rabelais, Tours 37200, France.; ^4^INSERM UMR-S1147, Paris 75006, France.; ^*^Both authors contributed equally.

**Keywords:** Tumor microenvironment, clear cell renal cell carcinoma, immune checkpoint inhibitor, immune checkpoint inhibitor resistance

## Abstract

The prognosis of metastatic clear cell renal cell carcinoma (mccRCC) has changed dramatically over the years with the emergence of immune checkpoint inhibitors (ICI) used alone, or in combination with another ICI, or with vascular endothelial growth factor receptor tyrosine kinase inhibitor. Although major response rates have been observed with ICI, many patients do not respond, reflecting primary resistance, and durable responses remain exceptional, reflecting secondary resistance. Factors contributing to primary and acquired resistance are manifold, including patient-intrinsic factors, tumor cell-intrinsic factors and factors associated with the tumoral microenvironment (TME). While some mechanisms of resistance are common to several tumor types, others are specific to mccRCC. Predictive biomarkers and alternative strategies are needed to overcome this resistance. This review provides an overview of the major ICI resistance mechanisms, highlights the potential of the TME to induce resistance to ICI, and discusses the predictive biomarkers available to guide therapeutic choice.

## Introduction

Kidney cancer accounts for 3%-5% of all cancers and is the seventh and tenth most frequently diagnosed malignancy amongst men and women respectively. Each year, about 330,000 new cases are diagnosed worldwide and 120,000 die from kidney cancer^[1]^. According to the 2016 OMS classification of renal tumors, clear cell renal cell carcinoma (ccRCC) is the most common (70%-90%), followed by papillary (10%-15%) and chromophobe RCCs (3%-5%). ccRCC is known to be insensitive to conventional chemotherapy. Until the 2000s, therapeutic options were limited and based upon high doses of the cytokines interleukin-2 (IL-2) and interferon, reflecting ccRCC sensitivity to immunotherapy. Almost 10% of patients treated with IL-2 achieved complete response but the safety profile was often limiting due to cardiac and respiratory toxic effects^[2]^. During the 2000s, a greater understanding of cancer biology led to increased therapeutic options. As a result, vascular endothelial growth factor receptor tyrosine kinase inhibitor (VEGFR-TKI) and mechanistic target of rapamycin inhibitors emerged as the new standards of care for metastatic clear cell renal cell carcinoma (mccRCC). VEGFR-TKIs sunitinib^[3]^ and pazopanib^[4]^ became the standard of care for untreated mccRCC. Temsirolimus and everolimus^[5]^ were available as first line treatment for poor-risk patients, according to the Memorial Sloan Kettering Cancer Center (MSKCC) criteria and after VEGFR-TKI failure. In recent years, the standard of care for mccRCC has changed dramatically with the emergence of immune checkpoint inhibitors (ICI) anti-programmed cell death 1 (PD-1) or anti programmed death ligand 1 (PD-L1), used as monotherapy or in combination with anti-cytotoxic T-lymphocyte-associated protein 4 (CTLA-4) or VEGFR-TKI. Nivolumab (a monoclonal antibody targeting PD-1) has been approved as second-line treatment for mccRCC^[6]^. Since 2018, new treatment combinations have emerged as first-line for patients with intermediate and unfavorable risk such as the nivolumab plus ipilimumab (targeting CTLA-4) combination^[7]^ and pembrolizumab (targeting PD-1) plus axitinib (Anti VEGFR-1, anti VEGFR-2, anti VEGFR-3 TKI) combination across all International Metastatic RCC Database Consortium (IMDC) prognostic risk groups^[8]^. Based on the JAVELIN Renal 101 trial, avelumab (targeting PD-L1) plus axitinib combination has also obtained the European Medicines Agency approval as first-line treatment of mccRCC across all IMDC prognostic risk groups irrespective of PD-L1 expression^[9]^.

Accordingly, the rationale of combining ICI and anti-VEGFR TKI is now well established. By reducing VEGF release and hypoxia, the antiangiogenic therapies have immunomodulatory effects. They reduce the proliferation of regulatory T cells (Treg), improve dendritic cell maturation and CD8+ T cells proliferation. By remodeling the vasculature, they also facilitate the penetration of immune cells into the tumor^[10]^.

With these growing therapeutic options, the question of therapeutic sequences to follow arises. To guide the clinician’s choice, predictive biomarkers are urgently required. ICI response biomarkers including tumor mutational burden and PD-L1 expression have failed to identify good responders in mccRCC. Genomic signatures are emerging as promising predictive biomarkers and could solve this major issue. Although ICI have dramatically changed the prognosis of mccRCC through major clinical response and overall response rates (42% with nivolumab-ipilimumab combination and 55% with pembrolizumab-axitinib combination respectively for untreated mccRCC, and 25% for nivolumab in second-line setting), a substantial number of patients do not respond, reflecting primary resistance to ICI. Disease progression rate approaches 100%, reflecting acquired resistance. Factors contributing to primary or acquired resistance are manifold, including patient-intrinsic factors, tumor cell-intrinsic factors and factors related to the tumoral microenvironment (TME).

We will highlight here the ICI resistance mechanisms, some of which are shared by most tumor types while others are specific to ccRCC. In particular, we will shed light on the TME potential to induce ICI resistance. We will also describe the available predictive biomarkers and discuss some recent approaches to overcome ICI resistance in mccRCC patients.

## Mechanisms of primary and secondary resistance to ICI

**Figure 1:**
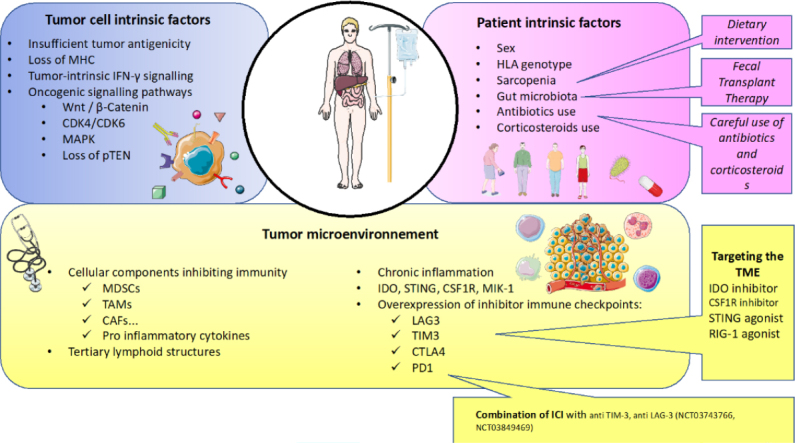
Main mechanisms of resistance to current immunotherapy in renal cell carcinoma. The main mechanisms of resistance to current immunotherapy can be divided into three major categories: tumor cell intrinsic factors, patient intrinsic factors and factors related to the tumor microenvironment. Here, some medical interventions to counteract these resistances are described. CAF: cancer associated fibroblasts; CDK4/CDK6: cyclin dependent kinase 4/cyclin dependent kinase 6; CSF1R: colony stimulating factor 1 receptor; CTLA4: cytotoxic T-lymphocyte antigen-4; CTK: cytokine; IDO: indoleamine 2 3-dioxygenase; IFN: interferon; HLA: human leukocyte antigen; ICI: immune checkpoint inhibitor; LAG3: lymphocyte-activation gene 3; MAPK: mitogen-activated protein kinase; MDSCs: myeloid derived suppressor cells; MHC: major histocompatibility complex; PD-1: programmed cell death 1; PTEN: phosphatase and TENsin homolog; RIG-1: retinoic acid-inducible gene 1; STING: stimulator of IFN genes; TAMs: tumor associated macrophages; TIM3: 1-5 T cell immunoglobulin mucin-3; TME: tumor Microenvironment

Factors contributing to primary or acquired resistance are manifold, including patient-intrinsic factors (such as sex, age, performance status, comorbidities, gut microbiota, human leukocyte antigen (HLA) genotype and genetic polymorphisms, use of antibiotics or steroids), tumor cell-intrinsic factors (such as tumor biology, tumor microenvironment, gene mutations or tumor mutational burden) and at the interface between the tumor and the host, the TME is undoubtedly one of the major players of immune and ICI resistance. Here, we will describe the main mechanisms of primary and secondary resistance to ICI occurring in cancer and specifically, in RCC [Figure 1]. For didactic purposes, resistance factors to ICI have been divided into those intrinsically related to the patient, the tumor and the microenvironment. In fact, all these factors interact with each other and have a final impact on anti-tumor immunity.

### Patient-intrinsic factors

#### Sex

Although sex-related dimorphism in the immune response is known, few studies have focused on the effect of gender on ICI efficacy. A recent meta-analysis by Conforti *et al*.^[11]^ included 11,351 patients with metastatic cancers, mostly melanoma and non-small cell lung cancer and revealed a significant difference in terms of overall survival (OS) between men and women (*P* = 0.0019), in favor of men^[11]^.

Almost 900 mccRCC patients enrolled in the Checkmate 025 trial (evaluating nivolumab *vs*. everolimus in second line setting or later) were included in this meta-analysis. Focusing on mccRCC patients, the hazard ratio (HR) for death was 0.84 (0.57-1.24) for women and 0.7 (0.5-0.9) for men, which was similar to the HR observed respectively in melanoma and non-small cell lung cancer patients.

Several hypotheses could explain this difference such as behavioral or biological factors. Firstly, exposure to mutagenic causative factors such as tobacco smoke or ultraviolet light without sun-protective measures is more frequent in men than in women. These behaviors are responsible for a greater increase in tumor mutational burden in men than in women. This has been reported in male patients’ tumors of different histiotypes^[11]^. Secondly, a role for estrogen modulation of the PD-1/PD-L1 axis has emerged from a few animal studies. Polanczyk *et al*.^[12,13]^ demonstrated that estrogen has effects on antigen-presenting cells and CD4+ CD25+ Foxp3+ regulatory T cells (Tregs): estrogen could up-regulate expression of FoxP3 and potentiate the regulatory activity of Tregs through the estrogen receptor alpha. Estrogen can also modulate PD-1 expression on dendritic cells, macrophages or Tregs^[12,13]^.

These findings need to be explored more deeply and the sex-dependent benefit of ICI must be confirmed in further prospective cohorts.

#### HLA genotype

In a recent study, Chowell *et al*.^[14,15]^ demonstrated that the functional diversity of the highly polymorphic human-leukocyte antigen class I (*HLA-I*) genes underlies immunologic control of cancer. They hypothesized that an *HLA-I* genotype with two alleles with sequences that are more divergent enables presentation of more immunopeptidomes, which may influence treatment response to ICI. Basically, heterozygous patients with more divergent alleles may present a larger set of peptides for T cell recognition than those with less-divergent *HLA-1* alleles. To test this hypothesis, they evaluated the germline *HLA-I* evolutionary divergence by quantifying the physiochemical sequence divergence between *HLA-I* alleles of each patient’s genotype. A high *HLA-I* evolutionary divergence was associated with a better response to ICI, both in melanoma receiving anti CTLA-4 or anti PD-(L)1, and in non-small cell lung cancer receiving anti PD-1. Median OS was significantly different between low and high *HLA-I* evolutionary divergence melanoma patients with almost 8 months *vs*. nearly 20 months (HR = 0.43, 95%CI: 0.2-0.8, *P* = 0.0094) respectively. Radiographic responses were also associated with mean *HLA-I* evolutionary divergence with 57% *vs.* 64% of clinical benefit in favor of high mean *HLA-I* evolutionary divergence [*P* = 0.003, odds ratio (OR) = 0.35]. Comparable results were observed in the non-small cell lung cancer cohort^[14,15]^. Till now, correlation between the functional diversity of *HLA-I* genes and immunologic control of cancer has not been specifically studied in mccRCC patients but this scientific concept can be extrapolated to other tumor types.

#### Sarcopenia

For a long time, sarcopenia has been known to be a negative prognostic marker in the pre-immunotherapy era^[16-18]^. In a recent retrospective multicenter real-life study, Cortellini *et al*.^[19]^ included 1000 consecutive advanced cancer patients (including 65% of non-small cell lung cancers, 19% of melanomas and 15% of mccRCCs) treated with ICI and focused on the putative association between sarcopenia (evaluated using cross-sectional image analysis on CT-scans, at the level of the third lumbar vertebra to evaluate skeletal muscle density and the skeletal muscle index) and clinical outcomes [objective response rate (ORR), progression free survival (PFS) and OS]. With a median follow-up of 20.3 months, patients with a low skeletal muscle index had a significant shorter OS (HR = 2.2, 95%CI: 1.3-3.6, *P* = 0.0026). Multivariate analysis confirmed skeletal muscle index as an independent predictor for OS. It should be noted that no association between skeletal muscle index and clinical response was identified, suggesting that sarcopenia may be prognostic rather than specifically predictive of response to ICI^[19]^.

#### Gut microbiota

Gut microbiota has been found to significantly impact the response to ICI in both mice and humans with epithelial tumors. Focusing on the metagenomics of stool samples from patients with lung and kidney cancers, Routy *et al*.^[20]^ revealed a positive correlation between the abundance of *Akkermansia muciniphila* and the response to ICI in mice receiving anti-PD-1 blockade. Besides, fecal microbiota transplantation from patients who responded to ICI into germ-free or antibiotic-treated mice improved ICI efficiency; in contrast, fecal microbiota transplantation from non-responding patients failed to do so. Oral gavage with *Akkermansia muciniphila* though, conferred responsiveness to ICI after fecal microbiota transplantation from non-responders. Fecal microbiota transplantation from responders caused accumulation of CXCR3+ CD4+ T cells in the tumor microenvironment and up-regulation of PD-L1 in splenic T cells. This established a cause-effect relationship between the anticancer efficacy of PD-1 blockade and the dominance of distinct commensal species. The dysbiosis may be associated both with malignant disease and concomitant antibiotic use. Following these preliminary results, a high number of translational studies have been launched and results are eagerly awaited.

#### Antibiotics and steroids use

As discussed above, dysregulation of gut microbiota may contribute to alter the systemic immune response. Antibiotic use seems to be a major cause of dysbiosis and leads to dysregulation of important commensal bacteria. A recent review^[21]^ summarized the currently available evidence addressing the impact of antibiotics on cancer patients receiving ICI. In 2018, Routy *et al*.^[22]^ focused on patients with non-small cell lung cancer, RCC and urothelial carcinoma, showing that antibiotic use was significantly associated, both in univariate and multivariate analyses, with reduced OS^[22]^. Regarding renal cell carcinoma, three retrospective studies confirmed this observation. Lalani^[23]^, Derosa *et al*.^[24]^ and Tinsley *et al*.^[25]^ all demonstrated a negative impact of antibiotics with a significantly PFS decrease of 5.5 months, 1.9 months and 2.7 months, respectively. Derosa and Tinsley also demonstrated a significant decrease in OS^[23-25]^. It should be mentioned that in these studies, multivariate analyses were adjusted for antibiotic-independent markers of poor prognosis (such as age, tumor type, recent hospitalization), suggesting that antibiotic use may be an independent prognostic factor.

Data is lacking about the consequences of antibiotic usage with regard to duration (short *vs.* long course), spectrum (broad spectrum combination *vs.* single-agent), timing (1, 2 or 3 months before ICI) and the time necessary for repopulation of the gut microbiota after discontinuation. More data is necessary to formulate guidelines for the optimal management of patients who require antibiotics before or during ICI treatment. It is interesting to note that many techniques used to modify an unfavorable gut microbiome into a favorable one are under investigation including fecal microbiota transplantation (NCT03341143), probiotics (NCT03637803, NCT03829111) and diet (NCT01716468).

Steroids are commonly used in oncology to treat routine symptoms such as dyspnea, fatigue, nausea or brain metastases. Steroid use with an equivalent prednisone dose ≥ 10 mg daily is known to induce immunosuppressive effects however, by impairing T cell activation, blocking Th1 expansion, favoring the recruitment of T regs, the expansion of M2 macrophages and altering the microbiota^[26]^. For these reasons, patients receiving steroids are commonly excluded from ICI clinical trials. Thus, data on ICI efficacy are lacking for these patients.

The impact of baseline steroids on ICI efficacy in patients with non-small cell lung cancer has been reported by Arbour *et al*.^[27]^. In this multicente (Gustave Roussy Cancer Center and MSKCC) retrospective analysis, baseline steroids use ≥ 10 mg daily was associated with worse outcomes with lower ORR (7% *vs.* 18%), lower PFS (*P* < 0.001) and OS (*P* < 0.001). In multivariate analysis of the pooled population, after adjusting for confounding factors, baseline steroid use remains significantly associated with worse outcomes. Interestingly, in the MSKCC cohort, patients who discontinued steroids 1 to 30 days before starting ICI had intermediate PFS and OS. It should be noted that on-treatment steroid use to manage immune-related adverse events does not negatively affect ICI efficacy^[28-31]^. Fucà *et al*.^[32]^ studied the modulation of peripheral blood immune cells by early use of steroids and its association with outcomes in patients with non-small cell lung cancer receiving ICI. No difference was found between patients receiving steroids at any time of the ICI course and never-exposed patients. By contrast, the early use of steroids (during the first month of treatment) was associated with low PFS (1.98 *vs*. 3.94 months, *P* = 0.0003) and OS (4.9 *vs*. 15.0 months, *P* < 0.001)^[32]^. To our knowledge, this issue has not been specifically studied in mccRCC patients.

### Tumor cell-intrinsic factors

#### Tumor biology

The Cancer-Immunity Cycle described by Chen and Mellman in 2013 summarizes the seven major steps of cancer immunity from antigen release to cancer cell destruction. The major steps are: (1) release of cancer cell antigens through cancer cell death; (2) cancer antigen presentation via antigen presenting cells; (3) priming and activation of antigen presenting cells and T cells; (4) trafficking of T cells to tumors; (5) infiltration of T cells into tumors; (6) recognition of cancer cells by T cells; and (7) cancer cell destruction. As described above, resistance can occur at each step of the Cancer-Immunity Cycle^[33]^. Consequently, the anti-tumor immune response results from interactions between tumor cells and their microenvironment.

#### Insufficient tumor antigenicity

The first reason why a tumor will not respond to ICI is the lack of recognition by T cells due to the absence of tumor neoantigens^[34]^. This is illustrated by the positive correlation between tumor mutational burden and ICI response across malignancies^[35]^. Another example is the high ICI response rate of patients harboring microsatellite instability due to mismatch repair defects. Special attention should be given to mRCC because no positive correlation between tumor mutational burden and ICI response was observed in this tumor type. In a small cohort of 34 patients, Labriola *et al*.^[36]^ showed that neither tumor mutational burden nor PD-L1 expression was correlated with patient outcomes or with ICI response^[36]^. Interestingly, Turajlic *et al*.^[37]^ showed that ccRCC tumors harbour the highest rate of insertion-deletion (indels) DNA alterations, leading to one of the highest neoantigenicity potential. This may be a strong rationale to explain ICI response among mccRCC patients^[37]^. Voss *et al*.^[38]^ showed that in two cohorts of mccRCC treated with anti-PD-1, a high frameshift count was associated with better OS (HR = 0.85; *P* = 0.006)^[38]^. Interestingly, in patients treated with TKI, this association was not significant (*P* = 0.07) and mutation as well as neoantigen burden did not have an impact on OS in patients treated with anti-PD-1 (HR = 1.02; *P* = 0.6; HR = 1.01; *P* = 0.69, respectively).

#### Tumor-intrinsic interferon gamma-signaling

An efficient T cell response against a tumor antigen depends on activation of the intrinsic interferon gamma (IFNγ) pathway within the microenvironment. As a result, Janus Kinase (JAK)-signal transducer and activator of transcription (STAT) is activated, leading to PD-L1 expression, through the activation of interferon regulatory factor 1 (IRF1). This adaptive expression of PD-L1 on the surface of tumor cells negatively regulates the anti-tumor T cell response. A genetic deficiency in IFNγ signaling pathways is associated with ICI inefficacy^[39]^. It should also be noted that IFNγ signaling enhances class I major histocompatibility complex (MHC) antigen presentation. In MHC deficient tumor-cells, pretreatment with IFNγ is required to express the antigen processing machinery and the class I MHC complex^[40]^. The IFNγ pathway also enables the recruitment of immune cells and has direct anti-proliferative and pro-apoptotic effects on tumor cells^[41]^. Zaretsky *et al*.^[42]^ revealed resistance-associated loss-of-function mutations in the genes encoding interferon-receptor-associated JAK1 or JAK2, concurrent with deletion of the wild-type allele. JAK1 and JAK2 truncating mutations resulted in a lack of response to IFNγ, including lack of response to its anti-proliferative effects on cancer cells^[42]^.

#### Pro-inflammatory cytokines

The RCC TME is associated with pro-inflammatory conditions. This is due to tissue damage that induces the release of pro-inflammatory molecules and cytokines such as adenosine triphosphate, IL-8, macrophage inflammatory protein 1-alpha, IL-6, tumor necrosis factor alpha (TNFα) or IFNγ. These cytokines recruit circulating leukocytes and have a pro-tumorigenic effect via the promotion of genomic instability, survival, cellular growth, angiogenesis and epithelial to mesenchymal transition. They also promote immunosuppression^[43]^. IFNγ induces increased expression of PD-1 on T cells and immune cells. The sustained expression of PD-1 and PD-L1 is responsible for T cell exhaustion, via the [Src homology 2 (SH2) domain-containing phosphatase 2] SHP2 recruitment. Transcriptional factors such as (signal transducer and activator of transcription 3) STAT-3 and (interferon regulatory factor 1) IRF1, induced by pro-inflammatory conditions, also modulate the expression of PDL1 and PDL2. IL-1, IL-6, IL-11, IL-17 and TNF alpha promote Treg expansion and increase T cell exhaustion^[44,45]^.

#### Regulation by oncogenic signaling.

Several signaling pathways have recently been identified as potential ICI resistance mechanisms. Here, we highlight three major oncogenic pathways among the most documented.

The Wnt/β-catenin pathway is involved in many biological processes from hematopoietic stem cell development, embryogenesis, and cell differentiation to immune regulation^[46]^. In most cancers, Wnt/β-catenin is overexpressed. Using human metastatic melanoma, Spranger *et al*.^[46]^ first demonstrated a correlation between activation of the Wnt/β-catenin pathway and absence of T cell gene expression signatures. Using mouse melanoma models, they also demonstrated that overexpression of Wnt/β-catenin is associated with T cell exclusion (resulting in “immune-desert” tumors) and resistance to anti-PD(L)-1 and anti CTLA-4 monoclonal antibody therapies^[46]^. This observation was confirmed in other tumor types including ovarian carcinoma, head and neck cancer, adenoid cystic carcinoma and urothelial carcinoma^[47-49]^. Wnt/β-catenin is also involved in the regulation of indoleamine 2,3-dioxygenase 1 (IDO1) and the peroxisome proliferator-activated receptor gamma (PPARgamma receptor), both inducing immunosuppressive effects^[50]^. A role in tumor stemness and dedifferentiation is also well-described^[51]^.

The mitogen-activated protein kinases (MAPK) pathway results mainly in VEGF, IL-6, IL-8 and IL-10 production and has known inhibitory effects on T cell recruitment and function. The MAPK pathway is also responsible for negative regulation of antigen presentation, MHC expression and reduced sensitivity to the anti-proliferative effects of IFNγ and TNFα^[39,52]^.

The comprehensive molecular characterization of ccRCC, led by The Cancer Genome Atlas Program, has identified the PI3K (phosphatidylinositol 3-kinase)/AKT (Protein Kinase B) pathway as one of the most currently altered pathways. The loss of Phosphatase and TENsin homolog (PTEN) is also a frequent molecular alteration^[53]^. The pathways activated after PTEN loss are responsible for poor T cell recruitment via activation of the autophagosome, and for a reduced type I interferon response to pathogen associated molecular patterns^[54]^.

Cyclin dependent kinase 4 and 6 (CDK4/6) and their co-factor D-type cyclins promote progression of the cell cycle from the G1 to S phase. In the last few years, four studies have underlined the impact of CDK4/6 inhibition on immune response. It was shown that the CDK4/6 inhibitor abemaciclib, in combination with anti-PD-L1 ICI, has a greater efficacy in mouse breast cancer models than either agent alone^[55,56]^. The positive effect of CDK4/6 inhibitors on antitumor immunity was attributed to their impact on T cells. Greater IL-2 production and increased T cell tumor infiltration were observed^[57]^.

#### Loss of MHC

The loss of class I and II MHC molecules can occur via multiple genetic and epigenetic mechanisms^[58]^, leading to the absence of recognition by cytotoxic T cells, resulting in immune-escape. Genetic and epigenetic mechanisms may potentially affect all the genes involved in the antigen processing and presenting machinery, particularly the gene of the B2-microglobulin (B2M) and thereby, MHC class I. Ribas’ team showed by whole-exome sequencing that a truncating mutation in the gene encoding for B2M led to loss of surface expression of MHC class I, which in turn resulted in loss of response to ICI in melanoma patients^[42]^. Sade-Feldman *et al*.^[59]^ also demonstrated that the loss of heterozygosity at the B2M locus was associated with lower OS in melanoma patients receiving ICI^[59]^.

### At the tumor-host interface: the tumor microenvironment as a major player of immune and ICI resistance

The bulk of the tumor is not limited to the tumor cells *per se* but include many other cells interacting with them within a cellular niche called the tumor microenvironment (TME), which sustains the growth of the tumor. The main cellular components of the TME are immune cells (including but not limited to natural killer (NK), T and B cells, macrophages, dendritic cells, *etc*.) and stromal cells (endothelial cells and fibroblasts). All these protagonists play an essential role in the development, progression and relapse of tumors. By combining these factors, the TME regulates the response to ICI and is a major target to overcome both primary and secondary ICI resistance. The composition and functional properties of the TME can be analyzed in order to find predicting profiles of ICI efficacy.

#### Main factors and their role in resistance to antitumor immune response

##### T cells

RCC is one of the most T cell-enriched tumors. Compared to other tumor types, the high densities of CD8+ tumor-infiltrating lymphocytes (TIL) is associated with a poor prognosis^[60,61]^. Many hypotheses underlie this contra-intuitive prognosis on the impact of CD8 in ccRCC: amongst them, it was demonstrated that CD8 TILs in ccRCC are mostly exhausted with frequent co-expression of PD-1 and lymphocyte-activation gene 3 (LAG-3), due to a lack of antigen presentation by dysfunctional/immature dendritic cells^[62]^. Until recently, the poor prognostic feature of CD8 TIL in ccRCC was controversial. Choueiri *et al*.^[5]^ reported exploratory data on CD8 infiltration from the randomized phase III trial JAVELIN RENAL 101, comparing avelumab (anti-PD-L1)-axitinib *vs*. sunitinib. High CD8 infiltration was associated with poor PFS for patients treated with sunitinib but not for patients treated with the avelumab-axitinib combination, suggesting that CD8 infiltration has prognostic value in ccRCC but loses it in patients treated with ICI. These data seem to be discordant with those coming from the NIVOREN-GETUG AFU 26 ancillary phase II study, where the highest CD8 infiltration in the invasive margin (scored 3 *vs*. 0-2) was associated with worse PFS (HR = 3.96, *P* < 0.0001) and OS (HR = 2.43, *P* = 0.04) for 324 patients treated with nivolumab. Nevertheless, only 7 of 324 patients were implicated and when comparing CD8 infiltrates between 0-1 and 2-3, no association was found with survival outcomes^[63]^. Moreover, CD8 density in the core of the tumor was not associated with outcomes (all *P* < 0.05 for PFS and OS). Interestingly, Voss *et al*.^[38]^ did not find any association between CD8 density and clinical benefit of ICI in two cohorts of mccRCC patients (*P* = 0.22)^[38]^.

In 2017, Giraldo *et al*.^[64]^ studied 40 primary ccRCCs. Resected tumors could be divided into three dominant immune profiles: (1) immune-regulated, characterized by polyclonal/poorly cytotoxic CD8+PD-1+ T cell immunoglobulin and Mucin-Domain Containing protein 3 (Tim-3)+ Lymphocyte-activation gene 3 (Lag-3)+ TILs and CD4+ Inducible Co-Stimulator (ICOS)+ cells with a Treg phenotype (CD25+CD127-Fox+3+/Helios+GITR+), that developed in inflamed tumors with prominent infiltration by dysfunctional dendritic cells highly expressing PD-L1; (2) immune-activated, enriched in oligoclonal/cytotoxic CD8+PD-1+Tim-3+TILs, that represented 22% of the tumors; and (3) immune-silent, enriched in TILs exhibiting RIL-like phenotype, that represented 56% patients of the cohort. Immune-regulated and immune activated tumors have a phenotypic signature that displayed aggressive histologic features associated with a high risk of disease progression, supporting the belief that they could benefit from ICI in combination with TME-modulating adjuvant treatment and closer clinical follow-up^[64]^.

Moreover, analysis of immune infiltration in RCC from the Cancer Genome Atlas showed a higher proportion of regulatory T cells in patients with a worse outcome (HR = 1.59, 95%CI: 1.23-0.06; *P* < 0.01). Mutated genes in this RCC cohort such as BIRC6, BAP1 and PIK3CA were associated with a higher fold-change in Tregs^[65]^.

##### Tumor associated macrophage

In 2011, a negative correlation between the anti-inflammatory macrophage phenotype (M2)(CD163+) and survival outcomes in RCC was identified^[66]^. In response to inflammatory stimuli, macrophages undergo M1 (classical) or M2 (alternative) activation. The M1 type produces high levels of inflammatory cytokines such as IL-12, IL-23 and IL-6. M2 macrophages can be subdivided into subsets called M2a, M2b, M2c and M2d^[67,68]^. The M2a phenotype is obtained by Th2 cytokine stimulation (IL-4, IL-13); activation of Toll-like receptors and immune complexes induce M2b; and IL-10 polarizes the M2c subtype. Tumor cells are able to switch the potential phenotype of macrophages into tumor-associated macrophages, which are characterized as the M2d subtype^[67]^, through the production of colony stimulating factor 1 (CSF1) for example. They express multiple receptors or ligands of inhibitory receptors such as PD-L1, PD-L2, B7-1. The presence of extensive tumor-associated macrophage infiltration into the RCC microenvironment contributes to cancer progression and metastasis by stimulating angiogenesis, tumor growth, cellular migration and invasion, as well as recruitment of Tregs to the tumor site by secreting CCL20 or CCL22. Therapeutic strategies have been suggested to suppress tumor-associated macrophage recruitment, to switch them back to the antitumor M1 phenotype^[69]^.

Surprisingly, Voss *et al*.^[38]^ recently reported that M2 macrophages, which were the most abundant infiltrating cell type, were associated with durable clinical benefit from anti-PD-1 therapy (*P* < 0.001). This association was not found in patients treated with TKI (*P* = 0.15)^[38]^. In the NIVOREN ancillary cohort, CD163 (M2 macrophages) with higher densities in the invasive margin was associated with better PFS (HR = 0.69, *P* = 0.016) but not OS (*P* = 0.5) in patients treated with nivolumab^[70]^.

##### B cells and tertiary lymphoid structures

The major role of B cells and tertiary lymphoid structures (TLS) in cancer biology has recently emerged. Five years ago, B cells with a regulatory role (also known as Bregs) were characterized as immunosuppressive cells secreting the immunosuppressive mediators IL-10 and transforming growth factor beta (TGFβ), which regulate Treg differentiation^[71]^.

A second population of more “classical” B cells has also been characterized within the tumor and in the stroma of metastases, defined by a strong memory response against tumor associated antigens^[72]^. Analysis from transcriptomic data of tumor samples, the Microenvironment Cells Populations-counter (MCP-counter), shows higher B cell related genes in a responder’s tumor as compared to non-responders in melanoma and ccRCC^[73]^. In sarcoma for example, patient clusters (SIC E) expressing high plasma cell signatures demonstrate an improved prognosis with anti PD-1 treatment^[74]^. TLS are lymph-node-like structures with a T cell zone with mature dendritic cells covering a follicular zone rich in proliferating and differentiating B cells as a germinal center. These structures are acting against tumor immunity and are associated with a good prognosis in patients with non-small cell lung, colorectal, breast, head and neck, pancreatic, and gastric cancers, RCC or melanoma. Depending on the maturation state and the cancer type (hepatocellular carcinoma), TLS can constitute a niche that favors the emergence of transformed cells and the development of activated Tregs^[75,76]^.

#### Hypoxia

RCC is one of the most hypervascular tumors composed of disorganized vessels. As such, nutrient and oxygen intake are insufficient, leading to hypoxia and a lower pH, both of which contribute to tumor progression^[77]^. Hypoxia induces the up-regulation of different genes involved in glucose metabolism, cell angiogenesis, cell proliferation, polarization of macrophages into Tumor Associated Macrophages, Treg recruitment and infiltration of myeloid derived suppressor cells leading to the inhibition of CD3+ T cells and the cytotoxic functions of CD8+ T cells^[78,79]^. Consequently, hypoxia-induced factor 1a and 2a release (HIF-1a and HIF-2a) induces increased expression of PD-L1 in tumor cells^[80,81]^. Hypoxia also enhances Treg abundance and NK-mediated antitumor immunity (synergized with B7-1) *in vitro* as well as *in vivo*^[82]^.

This high level of HIF-1 and HIF-2 mediates the generation VEGF^[82]^, which acts as an immune escape pathway by increasing the expression of the immune checkpoints CTLA4, TIM3, LAG3 on T cells and PD-L1 on dendritic cells^[79]^. Hypoxic tissues are enriched in adenosine deposits generated by CD39-CD37, which contribute to immune escape by suppressing the effect of T cells^[83]^.

#### Protein polybromo-1 expression and other immune checkpoints

Protein polybromo-1 (PBRM-1) is a major component of the polybromo-associated BAF (PBAF) form of the SWItch/Sucrose Non-Fermentable (SWI/SNF) chromatin remodeling complex. Inactivating mutations in this complex are common in a variety of cancers^[84]^. Using a clustered regularly interspaced short palindromic repeats (CRISPR) associated protein 9 (Cas9) screen, Pan *et al*.^[84]^ demonstrated that inactivation of PBRM1 sensitizes mouse melanoma cells to T cell killing. PBAF-deficient tumor cells produce more chemokines in response to IFNγ, promoting cytotoxic T cell functions^[84]^. Mutations in PBRM1 occur in 40% of ccRCC patients^[85]^. Using whole exome sequencing, Miao *et al*.^[86]^ demonstrated that the loss-of-function mutation in the PBRM1 gene was associated with clinical benefit in 35 mccRCC patients receiving ICI anti PD-1. PBRM-1 loss-of-function was enriched in tumors from patients experiencing a clinical benefit (*P* = 0.012). This was confirmed in an independent validation cohort^[86]^ and in a recent post-hoc analysis of archival tumor tissue from 382 patients enrolled in the Checkmate 025 phase III trial. PBRM1 mutations were associated with an improved PFS and OS in patients receiving nivolumab^[87]^. Vano *et al*.^[70]^ recently reported results on PBRM-1 protein status in 324 patients treated with nivolumab within the NIVOREN phase II trial (mentioned above). Better OS was found for patients with PBRM-1 loss with 12-month survival rates of 83.7% *vs.* 74% (*P* = 0.05). PBRM-1 loss was also associated with higher PD-1 and LAG-3 expression, and higher macrophage and CD8 T cell densities. Conversely, LAG-3 expression tended to be associated with a lower OS^[70]^. Nonetheless, inconsistent results have been observed. In the IMmotion 150 phase II study, PBRM-1 mutations were associated with improved PFS in the sunitinib group (HR = 0.38, 95%CI: 0.2-0.7). Atezolizumab monotherapy was associated with worse PFS compared to sunitinib in the PBRM1 mutant subgroup^[88]^.

In an analysis of CheckMate-010 phase II trial, CD8^+^PD-1^+^TIM-3^-^LAG-3^-^ tumor infiltrating cells were associated with a high level of T cell activation, a longer median immune related PFS and a higher immune related ORR to nivolumab^[89]^. PRBM-1 loss, TIM-3 and LAG-3 expression were all found in more aggressive mccRCC and could be therapeutic targets for ICI.

## Innovative approaches to overcome ICI resistances in mccRCC: ongoing trials and predictive tools

### Targeting the microenvironment

#### Indoleamine 2,3-dioxygenase 1 inhibitors

Indoleamine 2,3-dioxygenase 1 (IDO-1) upregulation is a key driver of T cell nutrient deprivation and can be targeted therapeutically. A short study on 15 mccRCC patients published in 2018 showed that IDO-1 overexpression (> 10%) in tumor endothelial cells was more frequent in case of response to nivolumab and associated with a better PFS. This would suggest that IDO could be used as a biomarker^[90]^.

At the ASCO 2018, Mitchell *et al*.^[91]^ reported the result of combination treatment with the oral IDO-1 enzyme inhibitor epacadostat and PD-1 inhibitor pembrolizumab. Activity in advanced solid tumors mainly concerned melanoma, non-small cell lung cancer and mccRCC, and was observed in the phase I/II ECHO-202/KEYNOTE 037 trial. An objective response occurred in 25 (40%) of 62 patients including 8 with complete responses and 13 with stable disease. Responses were observed for 2 patients with mccRCC out of 11^[91]^. These results lead to the phase III study, ECHO-301/KEYNOTE-252^[92]^, comparing epacadostat plus pembrolizumab *vs.* placebo in patients with unresectable or metastatic melanoma. In this trial, epacadostat failed to improve PFS or OS. The development of IDO-1 inhibitors was stopped after these negative results.

#### Colony stimulating factor 1 receptor inhibitor

As previously described, M2 macrophages promote tumor growth, angiogenesis, invasion, and metastasis as well as treatment resistance. Colony stimulating factor 1 receptor (CSF1R) expression allows the switching of type 1 macrophages into type 2 tumor-associated macrophages^[93]^. Combinations of CSF1R inhibitor and ICI are under investigation in phase I trials (NCT02718911, NCT02526017).

#### Stimulator of interferon genes and retinoic acid-inducible gene I agonists

Stimulator of interferon genes (STING) and retinoic acid-inducible gene I (RIG-1) are key mediators of nucleic acid sensing and thus, agonists targeting this two pathways are in early clinical development^[94]^. The STING pathway promotes IκB kinase and activates nuclear factor-kappa B and interferon regulatory factor 3, thus increasing production in pro-inflammatory cytokines^[95]^. RIG-1 contributes to the production and stimulation of immune cells, such as NK and CD8+ T cells^[96]^.

Two phase I trials are currently assessing STING agonist and RIG-1 agonist as monotherapy or in combination with ICI respectively in patients with metastatic solid tumors including mccRCC (NCT03010176 and NCT03739138).

### Targeting other immune checkpoints

#### TIM-3

As discussed above, TIM-3 and PD-1 co-expression on CD8 T cells is associated with worse outcomes including higher TNM stage, larger tumor size and lower PFS^[97]^. These findings argue for a therapeutic strategy targeting Tim-3 alone, or in combination with anti-PD-1/PD-L1. In 2018, there were four ongoing phase I trials assessing anti-TIM 3 antibodies: three in metastatic solid tumors and one in hematologic malignancies^[98]^. In 2019, another clinical trial assessing anti-TIM3 and anti-PD1 in combination has been open to patients with recurrent glioblastoma multiforme (NCT03961971). There has not been a clinical trial focusing on mccRCC to this day.

#### LAG-3

LAG-3 (also known as CD223) is another checkpoint identified on exhausted CD8 T cells^[64]^. Many clinical trials targeting PD-1 and/or LAG-3 in metastatic solid tumors including mRCC patients are ongoing^[99]^. In mRCC, a phase I clinical trial evaluating the safety of IMP321, a soluble LAG-3 Immunoglobuline fusion protein agonist, showed promising activity and acceptable toxicity^[100]^. Relatlimab, an anti-LAG3 antibody is under investigation, in combination with nivolumab in an ongoing phase II trial (NCT02996110). XmAb22841, a bispecific antibody targeting CTLA-4 and LAG-3, used alone or in combination with pembrolizumab, is evaluated in a phase 1 clinical trial for patients with select advanced solid tumors, including mccRCC (NCT03849469) [Tables 1-3].

**Table 1:** Major tumor cell intrinsic factors involved in ICI resistance mechanisms in ccRCC

Tumor cell intrinsic factor	Status	Consequences	Ref.
Interferon gamma-signaling	Activation	JAK-STAT pathway activation and PD-L1 overexpression	[38]
		Enhancement of class I MHC complex	[39]
		Recruitment of immune cells	[40]
Pro-inflammatory cytokines	High release	Genomic instability, promotion of tumor cells survival, angiogenesis	[44]
		mesenchymal to epithelial transition. Immunosuppression	
Wnt/β-catenin pathway	Over-expression	T cell exclusion, iregulation of IDO1 and PPARγ	[47,51]
PTEN	Loss of function	Inhibition of T cell recruitment	[55]
CDK4/6	Over-expression IL-2 production, increased T cells tumor infiltration	Tumor progression	[58]
MAPK pathway	Over-expression	Inhibition of T cell recruitment, negative regulation of antigen presentation	[38,53]

ICI: immune checkpoint inhibitors; ccRCC: clear cell renal cell carcinoma; JAK-STAT: Janus Kinase - signal transducers and activators of transcription; PD-L1: programmed cell death ligand 1; MHC: major histocompatibility complex; IDO1: indoleamine 2,3-dioxygenase 1; PPARγ: peroxisome proliferator-activated receptor gamma; PTEN: phosphatase and TENsin homolog

**Table 2:** Major tumor micro environment components involved in ICI resistance mechanisms in ccRCC

TME components	Status	Prognosis in RCC	Ref.
CD8+ T cells	High density	Poor	[61,63]
Regulatory CD4+ T cells	High density	Poor	[61]
Tumor associated Macrophages	High density	Poor	[61,67]
B cells	High density	Good	[61]
Tertiary Lymphoid Structure	High density	Good	[61,74]
Immune checkpoints and molecules of interest			
PBRM1	Loss of function	Good for pts receiving anti PD-1 nivolumab	[86,89]
LAG3	Overexpression	Poor	[89]
TIM3	Overexpression	Poor	[89]
PD-1	Overexpression	Poor	[89]
PD-L1	Overexpression	Poor	[89]

TME: tumor micro environment; ICI: immune checkpoint inhibitors; ccRCC: clear cell renal cell carcinoma; PBRM1: protein polybromo-1; PD1: programmed cell death 1; PD-L1: programmed cell death ligand 1; LAG3: lymphocyte-activation gene 3; TIM3: 1-5 T cell immunoglobulin mucin-3

**Table 3:** The most promising and innovative approaches to overcome such resistance

Targeted molecule	therapeutic combination	Results	Trial
IDO	Epacadostat (IDO1 enzyme inhibitor) + Pembrolizumab	40% objective response (62 total patient)	ECHO-202/KEYNOTE 037
	Epacadostat + Pembrolizumab or Placebo	Failed to improve OS or PFS	ECHO-301/KEYNITE-252
CSF1R	CSF1R inhibitors + ICI	Ongoing	NCT02718911/NCT02526017
STING	Inhibitor + Pembrolizumab	Ongoing	NCT03010176
RIG-1	Inhibitor + Pembrolizumab	Ongoing	NCT03739138
TIM3	No clinical trials focusing on mccRCC		
LAG3	Anti-LAG-3 antibody + ICI	Ongoing	NCT02996110
	Relatlimab (Anti-LAG-3) + Nivolumab	Ongoing	NCT02996110
LAG3 and CTLA4	XmAb22841 (bispecific) + Pembrolizumab or alone	Ongoing	NCT03849469

IDO: indoleamine 2,3-dioxygenase; CSF1R: colony stimulating factor 1 receptor; STING: stimulator of IFN genes; RIG-1: retinoic acid-inducible gene 1; TIM3: 1-5 T cell immunoglobulin mucin-3; LAG3: lymphocyte-activation gene 3; CTLA4: cytotoxic T-lymphocyte antigen-4; ICI: immune checkpoint inhibitors; PFS: progression free survival; OS: overall survival; mccRCC: metastatic clear cell renal cell carcinoma

### Predictive tools to guide therapeutic choices

In most tumor types, PD-L1 expression status alone is insufficient to determine which patients would benefit from ICI therapy^[101]^. Focusing on RCC, none of the ICIs need PD-L1 expression to be prescribed^[6-9]^. Current clinical, biological and histological markers such as the International Metastatic RCC Database Consortium (IMDC) score, Fuhrman grade, necrosis, vascular emboli or performance status are also imperfect in guiding our therapeutic decisions. Molecular signatures using next generation sequencing or cluster analysis are emerging as new, promising theragnostic assessments for immunotherapy in RCC. We will discuss here the main genomic signatures available in ccRCC.

Exploratory analyses were performed on the immune signatures assessed in the IMmotion 151 phase III trial comparing atezolizumab plus bevacizumab *vs.* sunitinib for mccRCC patients in the first line setting. A high angiogenesis signature was associated with an improved PFS with sunitinib. PFS was not improved by the combination of atezolizumab plus bevacizumab in the high angiogenesis signature group, whereas a significant benefit was observed in the low angiogenesis signature group. In the high T effector genes signature, PFS was improved by the combination of atezolizumab plus bevacizumab^[102]^. Choueiri *et al*.^[103]^ reported, at the 2019 ASCO congress, outcomes from biomarker analysis on tumor samples from the JAVELIN RENAL 101 Study (a phase III study assessing avelumab plus axitinib treatment for mccRCC patients in the first line setting). They assessed somatic mutations and analyzed relevant gene expression signatures (GES): effector T cells, angiogenesis, T cell-inflamed, and a novel immune-related signature incorporating pathway indicators for T and NK cell activation and IFNγ signaling, among others. PD-L1 expression (≥ 1% immune cells) was associated with the longest PFS in the avelumab plus axitinib arm, and the shortest in the sunitinib arm. Significant arm-specific treatment differences in PFS were observed relative to wildtype when mutations in genes such as CD1631L, PTEN or DNMT1 were present. Tumor mutational burden did not distinguish patients with respect to PFS and updated data will be presented^[103]^.

Ccrcc1-4 classification emerged in 2007 with the Tumor Identity Card program (“Carte d’Identité des Tumeurs” or CIT) for molecular characterization in solid tumors. Four molecular groups (ccrcc1 to 4) were identified: ccrcc1 = “c-myc-up,” ccrcc2 = “classical,” ccrcc3 = “normal-like” and ccrcc4 = “c-myc-up and immune-up”. Ccrcc4 subtype included frequent sarcomatoïd differentiation and high expression of markers of inflammation, such as members of the TNF and IRF families. Cytokine analyses revealed a strong expression of myeloid and T cell homing factors with their corresponding receptors and Th-1-related factors such as IFNγ and IL12. The immune suppressive IL10 and inhibitory receptors LAG3, PD-1, PD-L1 and PD-L2 were also highly expressed. In conclusion, the immune microenvironment in Ccrcc4 tumors is strongly inflammatory, Th1-oriented but immunologically suppressed. Rare mutations in VHL (Von Hippel Lindau) and PBRM1 were found in ccrcc4 tumors, but were frequent in ccrcc1 and ccrcc2 tumors without an effect on sunitinib response. The ccrcc1/ccrcc4 tumors represented 76% of Fuhrman grade 4 compared with 56% in ccrcc2/ccrcc3 tumors, suggesting a less differentiated stem-cell phenotype. The ccrcc2 subtype was not characterized by specific pathways and showed an intermediate expression signature between ccrcc3 and ccrcc1/ccrcc4-related profiles^[104]^.

BIONIKK is an ongoing multicentric molecular-driven randomized phase 2 trial (NCT02960906). This is the first trial studying the personalization of treatment according to tumor molecular characteristics in mccRCC. Using a 35-gene expression mRNA signature, patients were divided into four molecular groups (1 to 4). Patients in groups 1 and 4 were randomized to receive nivolumab alone (arms 1A and 4A) or nivolumab plus ipilimumab for four injections followed by nivolumab alone (arms 1B and 4B). Patients in groups 2 and 3 were randomized to receive nivolumab plus ipilimumab followed by nivolumab alone (arms 2B and 3B) or a tyrosine kinase inhibitor (sunitinib or pazopanib at the investigator’s choice (arms 2C and 3C)^[105]^. The main objective is to evaluate the response rate by treatment and molecular group.

## Conclusion

The prognosis of mccRCC patients has changed dramatically over the past few years. Impressive response rates including complete responses have been observed with ICI, used either as monotherapy or in combination with other ICIs or anti-angiogenic therapies. Many patients do not respond however, reflecting primary resistance and durable treatment response remains rare with resistance rates approaching 100%. ICI resistance mechanisms are manifold including patient-intrinsic factors, tumor cell-intrinsic factors and factors associated with the TME. Many innovative approaches to overcome ICI resistance are under investigation in phase I clinical trials on mccRCC. Due to the emergence of new study methods, the TME composition and its role in promoting tumor and therapy resistance is increasingly better understood. The TME molecules such as IDO-1, CSF1R, STING, RIG-1 and the immune checkpoints TIM-3 and LAG-3 are possible areas for future research. Besides these new therapeutic combinations, genomic signatures are emerging as exciting predictive biomarkers to guide treatment choice and overcome primary resistance. Results of the ongoing phase II clinical trial BIONIKK are awaited to, hopefully, bring new insights on this challenging field.

## Declarations

### Copyright

© The Author(s) 2020.
